# Gut microbiota fingerprinting as a potential tool for tracing the geographical origin of farmed mussels (*Mytilus galloprovincialis*)

**DOI:** 10.1371/journal.pone.0290776

**Published:** 2023-08-30

**Authors:** Ane del Rio-Lavín, Sébastien Monchy, Elisa Jiménez, Miguel Ángel Pardo

**Affiliations:** 1 AZTI, Food Research, Basque Research and Technology Alliance (BRTA), Derio, Bizkaia, Spain; 2 LOG, Laboratoire d’Océanologie et de Géosciences, Univ. Littoral Côte d’Opale, CNRS, Univ. Lille, UMR 8187, Wimereux, France; Laboratoire de Biologie du Développement de Villefranche-sur-Mer, FRANCE

## Abstract

Identifying the provenance of seafood is critical to combat commercial fraud, enforce food safety regulations and ensure consumers’ confidence. Hence, the current study aimed to determine if the bacterial composition present in the digestive gland and stomach of *M*. *galloprovincialis* mussels could be used as traceability approach to discriminate their geographic origin. The microbiota of 160 mussels collected seasonally in 2019 from five different mussel farms located in three regions in Spain (Galicia, Basque Country and Catalonia) was characterized using 16S rRNA targeted amplicon sequencing. Results showed that the bacterial community composition/fingerprint was significantly different between harvesting locations and seasons, with the effect prompted by the origin exceeding the seasonal variability. To further evaluate the stability and potential of this traceability approach, the bacterial fingerprint of 20 new individuals collected from the Basque Country in autumn 2020 were compared to the profiles obtained in 2019. Results showed that mussels collected from the Basque Country in two consecutive years cluster together, even matching the season of harvesting. The findings of this preliminary study support that this methodological approach has the potential to trace the geographical origin of unprocessed mussels and could have potential uses in seafood traceability and food safety.

## Introduction

Fisheries and aquaculture production is at record high and provides nutritious food and employment options to many countries around the world. Global production of aquatic animals reached 214 million tonnes in 2020, valued in 406 billion USD [1]. As the demand for fish and fish products increases, so does the awareness of the need to ensure transparency and traceability along the food chain. In the EU, the Council Regulation (EC) N° 1379/2013 on the common organization of the markets in fishery and aquaculture products, requires that seafood labelling indicates the commercial designation and scientific name of the species, the production method, and the area where the product was caught or farmed [2]. Thus, tracing the geographic origin of seafood represents a major goal for control authorities to address current legislation, but also to prevent commercial fraud and to ensure sustainable fisheries and aquaculture management. Traceability is also essential to protect public health by preventing hazardous products from reaching the marketplace. Consuming seafood, and specially shellfish, from restricted areas which may be contaminated with biotoxins, bacterial pathogens or chemical pollutants, poses a potential health risk for consumers. Likewise, being able to guarantee the provenance is also relevant for producers who aim at certifying their products and promote consumers’ confidence. In this context, a global need for suitable geographic origin traceability analytical tools has emerged.

Several experimental approaches have been proposed to validate the geographic origin of seafood, which include techniques based on DNA markers, fatty acids, trace elemental profiling and stable isotope analysis [3–5]. Another methodology that has also been considered is the analysis of the microbial communities associated to seafood and its linkage to a particular geographic area. In this regard, the potential of analysing the bacterial composition by 16S rRNA targeted Next-Generation Sequencing (NGS) has been explored to trace the geographic origin of cultured seabass (*Dicentrarchus labras*) [6], Manila clams (*Ruditapes philippinarum*) [7] and soft-shell clams (*Mya arenaria*) [8].

In this study, we wanted to assess the applicability of this bacterial profiling approach to differentiate the origin of the highly commercialized *Mytilus galloprovincialis* mussel. In the European Union (EU), mussels represent a major aquaculture species accounting for 34% of the total production. Spain is the main producer of *M*. *galloprovincialis* in the EU, reaching 247,897 tonnes in 2020 for a market value of 147 million euro. More than 97% of the Spanish mussels’ production is carried out in Galicia, a region located in the north-western coast of the Iberian Peninsula, and whose mussels fall within the domain of Protected Designation of Origin (PDO) “Mexillón de Galicia”. Thus, this geographic area represents the perfect scenario to perform a preliminary study that evaluates the suitability of using bacterial communities present in mussels’ digestive gland and stomach to validate their origin. Indeed, the microbiota of filter-feeding bivalves has been shown to be influenced by the geographic location, but also by season, temperature, salinity and other environmental conditions [9–14].

Therefore, the present study aimed to assess whether the geographic origin influences the bacterial composition present in the digestive gland and stomach of *M*. *galloprovincialis* mussels in such way that could be used as a traceability approach. First, the effect of geographical origin and seasonal variations has been examined by analysing the gut microbiota of mussels collected seasonally from five different farms in Spain using 16S rRNA targeted NGS. Then, the potential and stability of this traceability approach has been evaluated by attempting to trace the geographical origin of new samples collected a year after.

## Materials and methods

### Sampling and DNA extraction

**Fig 1:**
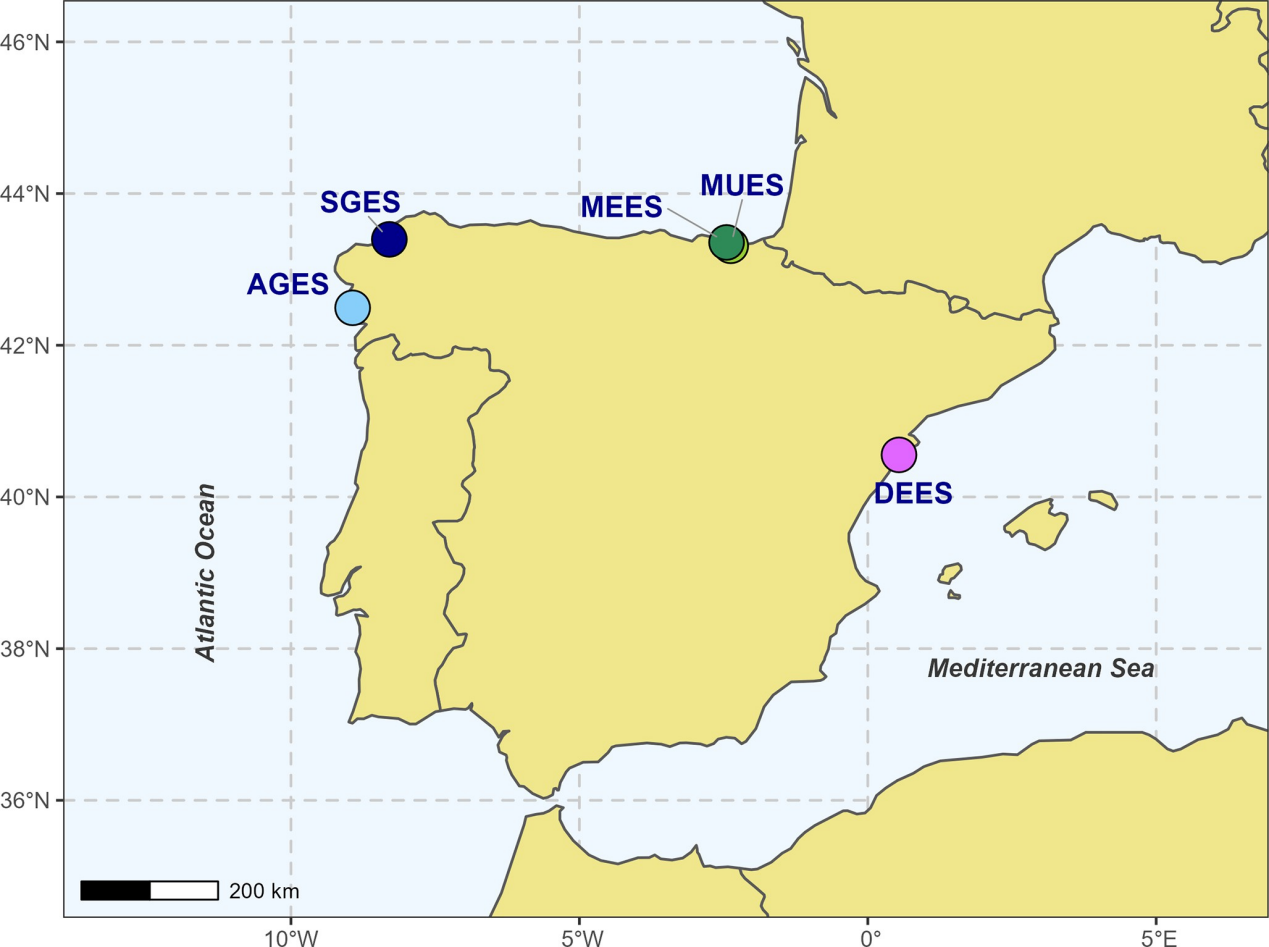
Map showing the geographic location where the samples used for this study were collected. Samples were collected from three different regions: Galicia [Ría de Arousa (● AGES) and Ría de Betanzos-Sada (● SGES)], Catalonia [Delta Ebro (● DEES)] and Basque Country [Mendexa (● MEES) and Mutriku (● MUES)].

Mussel samples were collected seasonally from five different farms in Spain during 2019: Mutriku (MUES; Basque country; rafts within a small port), Mendexa (MEES; Basque country; offshore longlines), Ría de Arousa (AGES; Galicia; rafts in estuarine inlets), Ría de Betanzos-Sada (SGES; Galicia; rafts in estuarine inlets) and Delta Ebro (DEES; Catalonia; offshore longlines) (Fig 1) (S1 File). A total of 160 *M*. *galloprovincialis* adult mussels (n = 8 per location/season) were collected and immediately transported to the laboratory on ice. To further evaluate the stability of the bacterial fingerprint, 20 additional mussel samples (10 from each farm) were collected from the Basque country farms a year later (September 2020) (S1 File). In the laboratory, mussels were scrubbed to remove epibionts and gently washed with artificial seawater (ASW) to remove part of the non-resident microbiota. For each mussel, the digestive gland and stomach (DGS) was removed under sterile conditions and immediately stored at −80°C. Bacterial DNA extraction was performed using the DNeasy PowerSoil Kit (Qiagen, Germany), starting from 50 mg of the DGS and following manufacturer’s instructions. Extracted DNA concentration was determined by means of the Quant-iT dsDNA HS assay kit using a Qubit® 1.0 Fluorometer (Life Technologies, USA). As a control, surface seawater samples (2L per location/season) were also collected (see S2 File)”.

### Library preparation and next generation sequencing

The 16S amplicon library was prepared according to a standardized Illumina protocol [15]. Briefly, the V3-V4 region of bacterial 16S ribosomal RNA gene was amplified by using universal primers 341F (5′- CCTACGGGNGGCWGCAG-3′) and 805R (5′-GACTACHVGGGTATCTAAT CC-3′) [16] tagged with 33 bp overhang adapters. These PCR reactions (15 μl) contained an initial concentration of 0.2 μM of each primer, 7.5 μl of Phusion High-Fidelity PCR Master Mix with HF Buffer (1X) (Thermo Scientific, USA) and 20 ng of template DNA. The PCR cycles started with a 3 min initial denaturation at 98°C, followed by 30 cycles of 30 s at 98°C, 30 s at 55°C and 30 s at 72°C and a final 10 min elongation at 72°C. Triplicate PCR reactions were pooled and purified using AMPure XP beads (Beckman Coulter, USA) according to the manufacturer’s instructions. Secondary PCR reactions were performed in order to add Nextera XT Illumina sequencing indices and adapters. The reaction conditions were 3 min at 98°C, followed by 8 cycles of 30 s at 98°C, 30 s at 55°C and 30 s at 72°C, and a final 10 min elongation at 72°C. After purification, 16S amplicon libraries were pooled together at equimolar concentration and subjected to Illumina MiSeq paired-end sequencing (Illumina, USA). The 16S amplicon library of mussel samples collected in 2020 was prepared and sequenced in a different batch. All sequencing data has been submitted to the NCBI sequence read archive database (BioProject accession: PRJNA899701).

### Sequence processing

Sequences were analysed using Mothur software v.1.44.3 [17] following the standard operating procedure (http://www.mothur.org/wiki/MiSeq_SOP). Sequences were quality filtered, de-replicated to unique sequences and aligned against the SILVA database [18]. Suspected chimeras were removed using Uchime software [19]. Resulting sequences (median length of 460 bp) were clustered into Operational Taxonomical Unit (OTU) using 97% similarity threshold. For taxonomic affiliation, OTU sequences were searched against the latest available SILVA database (Release 138.1) using the Wang approach [20] as implemented in Mothur. OTUs corresponding to chloroplasts and mitochondria were removed from the data set.

### Bioinformatics and statistical analyses

Alpha diversity estimators (observed OTUs, Shannon, Simpson and Berger Parker) were calculated using rarefied data (by random picking, based on the sample having the lowest read number) with Past 3.26 software [21] for each sample. All other statistical analyses were performed in R [22]. Differences in alpha diversity patterns between sampling points and seasons were analysed using ANOVA and Tukey’s honest significance test of ANOVA (pairwise comparisons) for normally distributed metrics and Kruskal-Wallis and Wilcoxon rank sum test (pairwise comparisons) for non-parametric metrics (“stats”package). Normality and homoscedasticity of each metric were tested using Shapiro-Wilks and Fligner-Killeen tests (“stats”package) respectively.

To assess beta diversity, sample-specific read abundances of OTU were subjected to Hellinger-transformation using the function ‘decostand’ (method = ‘hellinger’, which consist of dividing the total reads for each OTU by the total number of reads in the corresponding sample and taking the square root of the quotient). All subsequent analysis were based only on standardized data. The variation in microbiota composition between samples based on Bray-Curtis dissimilarities (at OTU level) was visualized by hierarchical cluster analysis using ‘hclust’ function (“stats”package) and by non-metric multidimensional scaling (NMDS) using ‘metamds’ function (“vegan” package). Differences in beta diversity between sampling points and seasons were tested using a permutational multivariate analysis of variance (PERMANOVA) using ‘adonis’ function (“vegan” package). Statistical significance was determined based on adjusted p-values after the Benjamini-Hochberg correction [23] and pairwise comparisons were performed using ‘pairwise.adonis’ function (“vegan”package). Homogeneity of variance was evaluated with the ‘betadisper’ function (“vegan” package).

For taxon distribution analysis, relative abundances were calculated at each taxonomic rank for all datasets. Individuals were grouped by location and season, and the top 20 taxa of each subset visualized using the R “ggplot2” package. Based on the relative abundance, taxa significantly different between harvesting regions was determined by Kruskal-Wallis and Wilcoxon rank sum test (pairwise comparisons) with Benjamini-Hochberg FDR correction of the p-value. Finally, to evaluate the stability and the potential of this approach to trace the origin of new samples, a taxonomy based hierarchical NMDS analysis was performed combining 2019 and 2020 bacterial profiles.

## Results

### Gut microbial community structure of mussels from different geographic origins and harvesting seasons

Overall, a total of 8,001,734 bacterial sequences were obtained by Illumina MiSeq high-throughput sequencing targeting the V3-V4 region of the 16S rRNA gene, with an average of 42,279 reads per mussel gut sample and 18,158 reads per seawater sample. These sequences were assigned to a total of 115,514 OTUs with a 3% level of genetic dissimilarity.

**Fig 2:**
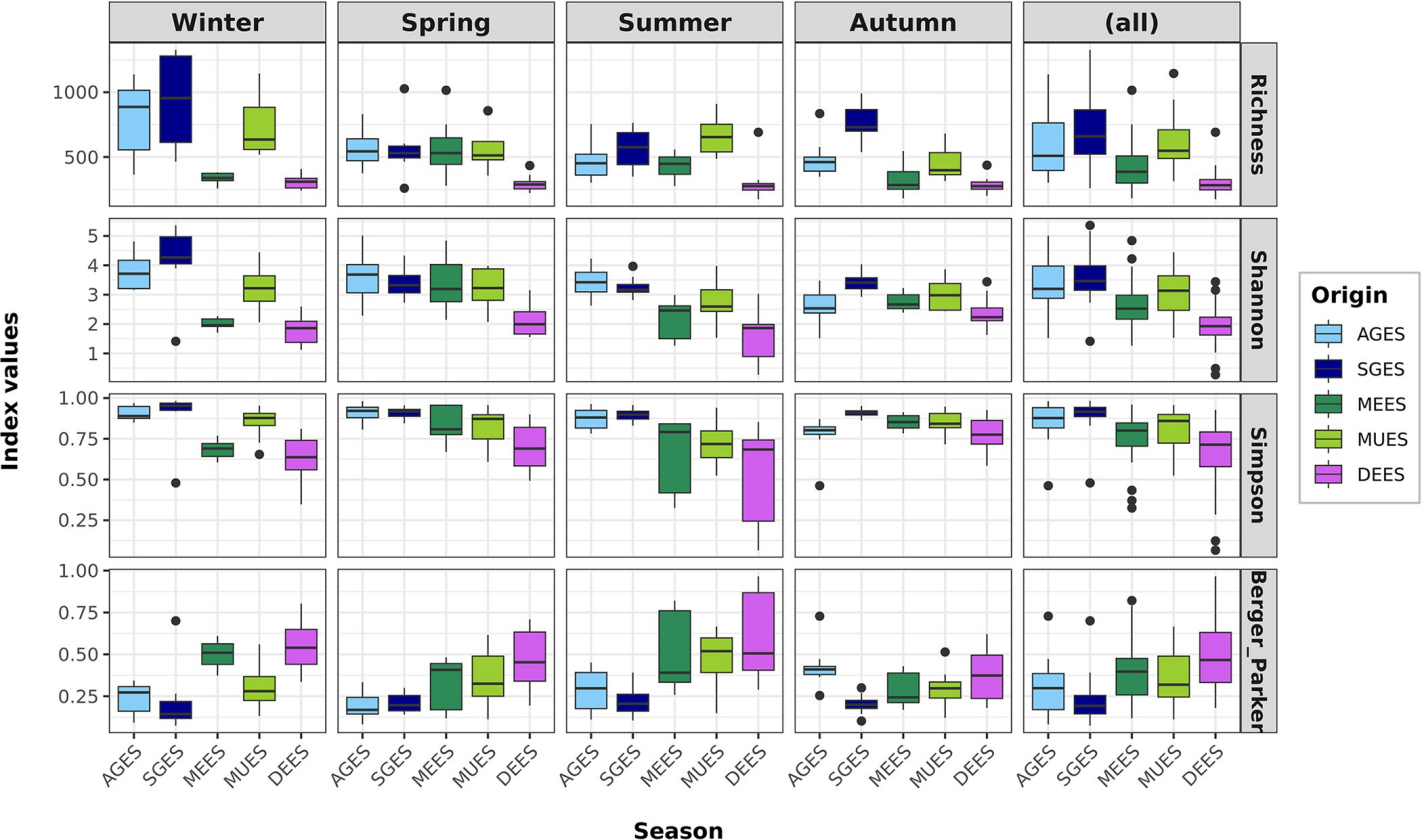
Alpha diversity of *M*. *galloprovincialis* DGS microbiota. The box plot shows mean values and standard deviation of the richness, diversity (Simpson and Shannon) and dominance (Berger-Parker) estimators for bacterial communities according to the season and harvest locations: in Galician region (● AGES, ● SGES), Catalonia region (● DEES) and Basque Country region (● MEES, ● MUES).

For alpha diversity analysis, each mussel sample was rarefied to 16,963 bacterial reads, resulting in a total of 42,062 OTUs (18,294 OTUs with counts ≥2), with an average of 581, 705, 604 (621 in 2020), 421 (702 in 2020) and 302 OTUs for locations AGES, SGES, MUES, MEES and DEES respectively. Results revealed that richness, diversity (Shannon and Simpson) and dominance (Berger Parker) estimators were significantly (Kruskal-Wallis, p<0.05) influenced by the sampling location of mussels and the seasonality (Fig 2 and S3 File). Higher bacterial richness and diversity, and lower dominance were observed in mussel farms located in rafts in estuarine inlets (AGES and SGES) and ports (MUES), compared to mussel farms located in offshore longlines (MUES, DEES), with significant differences between those inshore and offshore locations (Wilcoxon rank sum test, p<0.05) (S4 File). Regarding seasonality, winter period displayed the highest differences in mussel gut microbial alpha diversity between farming locations, whereas autumn had the lowest.

**Fig 3:**
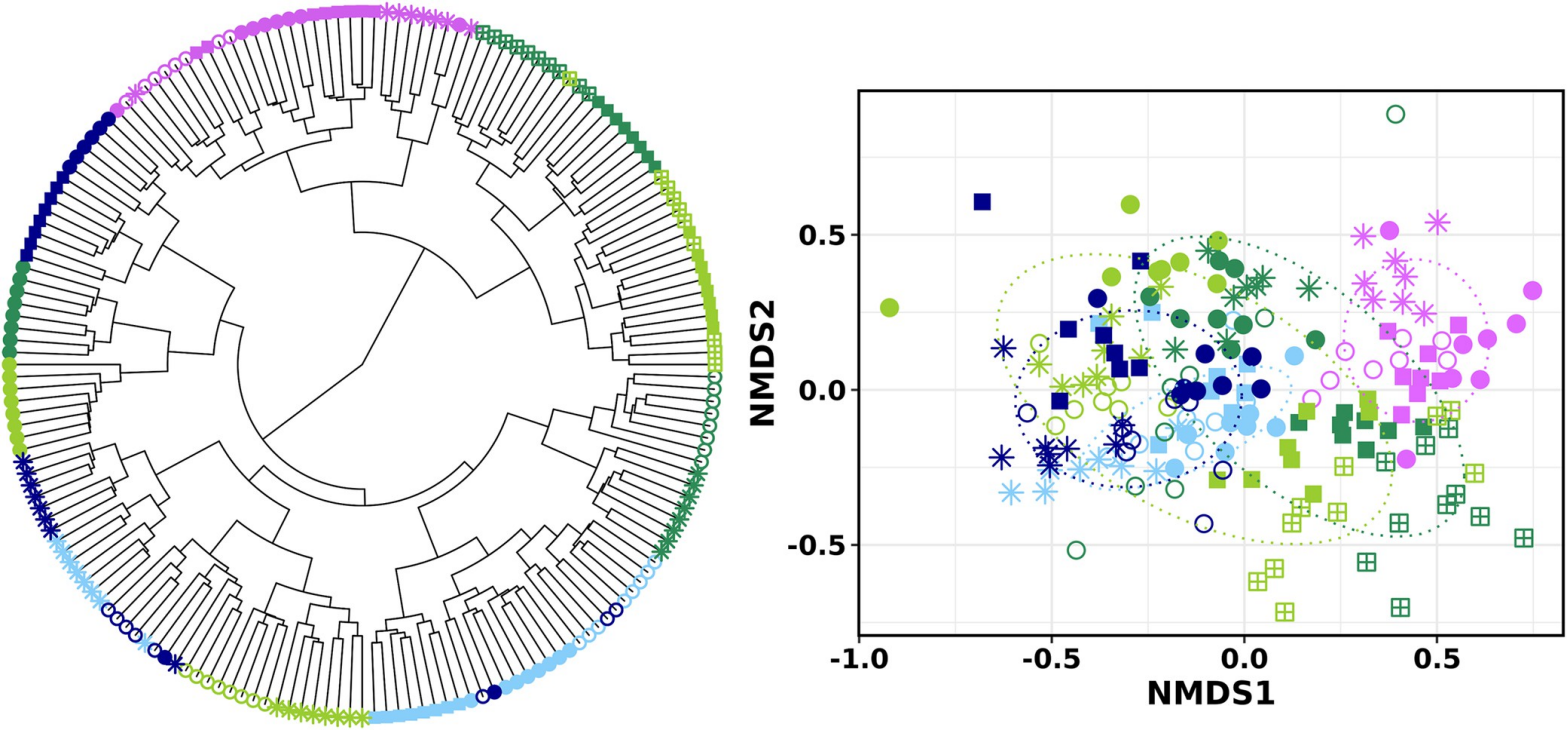
Hierarchical clustering dendrogram (**left**) and NMDS ordination (**right**) based on Bray-Curtis dissimilarities at OTU level of farmed mussel gut microbiota. Each symbol represents an individual *M*. *galloprovincialis* mussel; shapes of symbols correspond to different harvesting seasons—winter (✳), spring (◯), summer (●), autumn 2019 (◼), autumn 2020 (Σ)—and colours correspond to different harvest locations in Galician region (● AGES, ● SGES), Catalonia region (● DEES) and Basque Country region (● MEES, ● MUES).

Regarding beta diversity, hierarchical clustering dendrograms and NMDS ordination based on Bray-Curtis dissimilarity matrix revealed that gut bacterial sequences were grouped first by mussel origin and then by season (Fig 3). PERMANOVA results confirmed this observation, with farm geographical origin (by site, five levels) (P = 0.000999, R2 = 0.129, F = 6.549) and harvesting season (P = 0.000999, R2 = 0.063, F = 3.946), as well as the interaction between these two factors (P = 0.000999, R2 = 0.154, F = 3.134), contributing significantly to the variation observed among the microbial communities. The geographic origin effect was also significant across all four seasons (p<0.05) when each season was analysed individually (S5 File). Results showed a clear separation between the microbial community structure of mussels collected from Galician (AGES, SGES) and Catalonian farms (DEES). However, Basque Country samples (MEES, MUES) grouped distinctly depending on the season. The microbiota of mussels collected from the Basque Country region in autumn were closer to the Catalonian fingerprint, whereas winter and spring Basque country microbial structures were closer to those from Galicia. Significant differences were still depicted when performing the statistical analysis at a regional scale (Galicia, Catalonia and Basque Country) instead of by farm (P = 0.000999) (S6 File). Regarding seasonality, multiple pairwise comparisons between seasons performed separately for each sampling site, showed that the DGS bacterial communities differed significantly between all four seasons (pairwise PERMANOVA, p<0.001) (S7 File) when the five mussel farms were analysed individually.

### Potential of using mussel microbiota as geographic indicator

#### Identification of taxa that differ between origins

Once settled that mussel DGS microbiota differed depending on their geographical region, farming site and harvesting season, we wanted to identify which taxa had a significantly different representation between the geographical regions and could therefore act as potential origin indicator.

Overall, the bacterial community of mussel DGS consisted of 19 phylum, 86 families and 109 genus (> 0.1% relative abundance). The seven most abundant bacterial phyla, which collectively accounted for 88.33% of all reads, were: *Proteobacteria* (32.18%), *Firmicutes* (23.40%), *Bacteroidota* (13.85%), *Planctomycetota* (5.68%), *Verrucomicrobiota* (5.45%), *Cyanobacteria* (3.34%), *Fusobacteriota* (2.37%) and *Actinobacteriota* (2.02).

**Fig 4:**
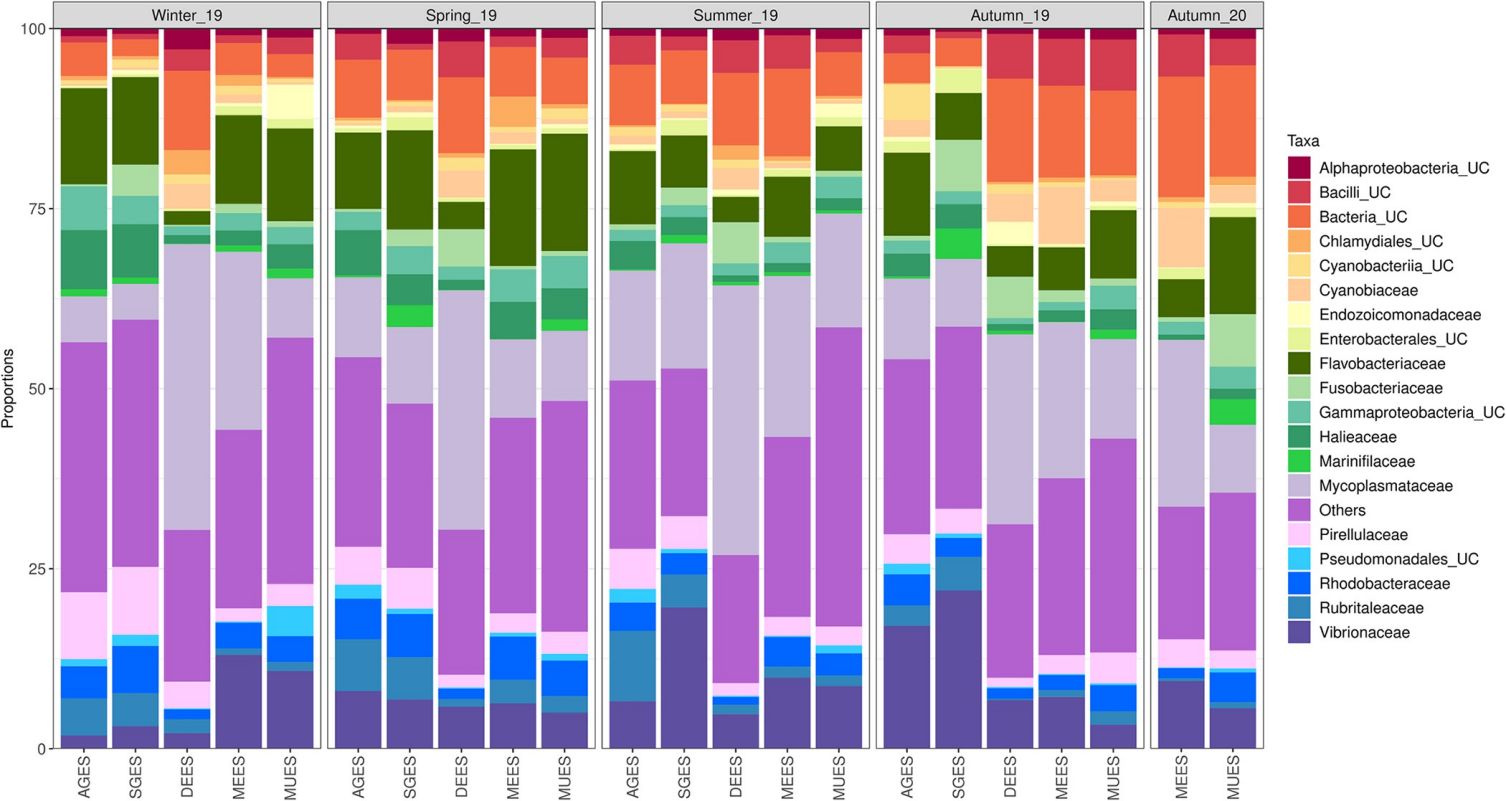
Average relative abundances of bacterial communities, at family level, of mussel DGS harvested from five different farms: In Galician region (AGES, SGES), Catalonia region (DEES) and Basque Country region (MEES, MUES). Taxa not within the 20 most abundant families were pooled together as “Other”. UC = unclassified.

At family level, bacterial community composition of mussel DGS differed between geographic locations and seasons (Fig 4). According to Wilcoxon rank sum test analysis based on bacterial relative read abundance, several taxa displayed significantly different representation between the three geographical regions. Overall, mussels from the Catalonian region showed significantly lower relative abundances of *Vibrionaceae*, *Rhodobacteraceae* and *Rhizobiaceae* (phylum *Proteobacteria*) and *Flavobacteriaceae* (phylum *Bacteroidetes*), with respect to the other two regions. Mussel farms from the Galician region showed higher proportion of *Rubritaleaceae* (phylum *Verrucomicrobiota*), *Pseudomonadales* (phylum *Proteobacteria*) and *Ilumatobacteraceae* (phylum *Actinobacteriota*). Finally, mussels from the Basque Country region showed higher proportion of *Cyclobacteriaceae* (phylum *Bacteroidota*) with respect to the other two regions.

**Fig 5:**
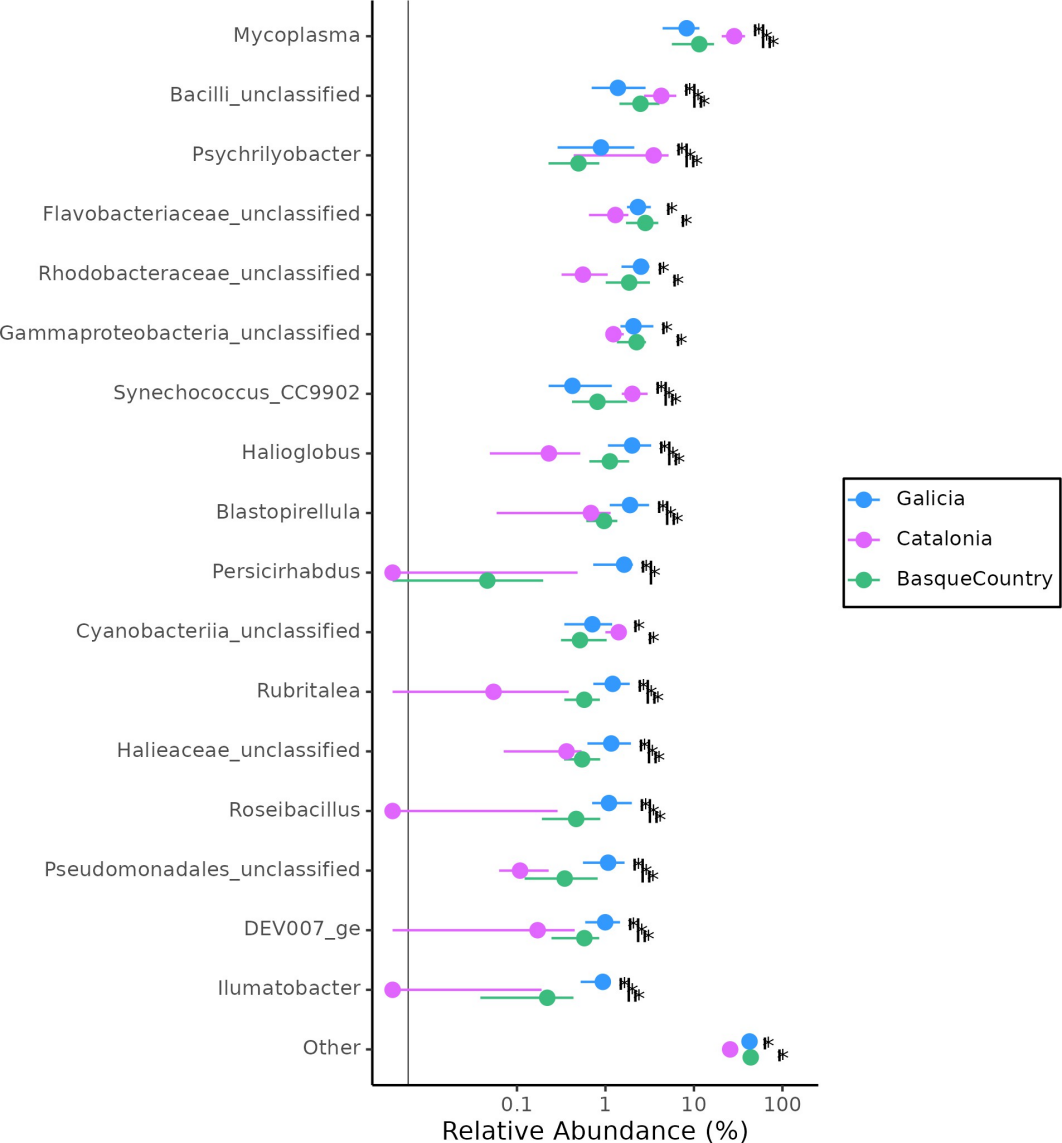
Relative abundance of genus that differed significantly (Kruskall Wallis, p<0.05) between the three mussel farming regions. Points are median values; lines represent the interquartile range and the black vertical line is the limit of detection. Pairwise comparisons were calculated by Wilcoxon rank sum test with Benjamini-Hochberg correction and significant differences are marked with asterisks (*). Taxa with a median relative abundance < 0.5% for all the locations were grouped in “Others”. Colours correspond to different harvest regions: Galician region (● AGES + SGES), Basque Country region (● MEES + MUES) and Catalonia region (● DEES).

At genus level, mussels from the Catalonian region showed significantly lower relative abundances of *Rubritalea* (*Rubritaleaceae*), *Roseibacillus* (*Rubritaleaceae*), *Ilumatobacter* (*Ilumatobacteraceae*) genus with respect to the other two regions (Fig 5). Galician region showed a significantly higher proportion of *Persicirhabdus* (*Rubritaleaceae*). However, when applying these analyses on farming site (S8 File), predominant genus also differed within the same region.

### Stability of the bacterial community composition

Another critical factor, that would determine whether the bacterial community composition of mussels could be used as a reliable origin traceability tool, is the stability of the fingerprint associated to each farm. To assess whether the microbial composition changed every year, the bacterial community structure of samples collected in 2019 was compared to those collected, sequenced and analysed independently a year later (2020) in the Basque Country at the exact same locations (MEES and MUES).

First, we evaluated the consistency and robustness of potential bacterial candidates which showed a significantly different representation by origin and could act as geographic origin indicators. Although some genera, such as *Persicirhabdus*, showed similar proportions in 2020 (S8 and S9 Files), others such as *Rubritalea*, *Roseibacillus* and *Polaribacter* showed significantly lower values a year later, closer to the values expected for Catalonia. This means that all the potential markers were not entirely consistent one year later and thus, focusing on specific differences (a small group of taxa or of OTUs that have significantly different representation between the geographical regions) might not be a suitable and robust approach for origin traceability.

**Fig 6:**
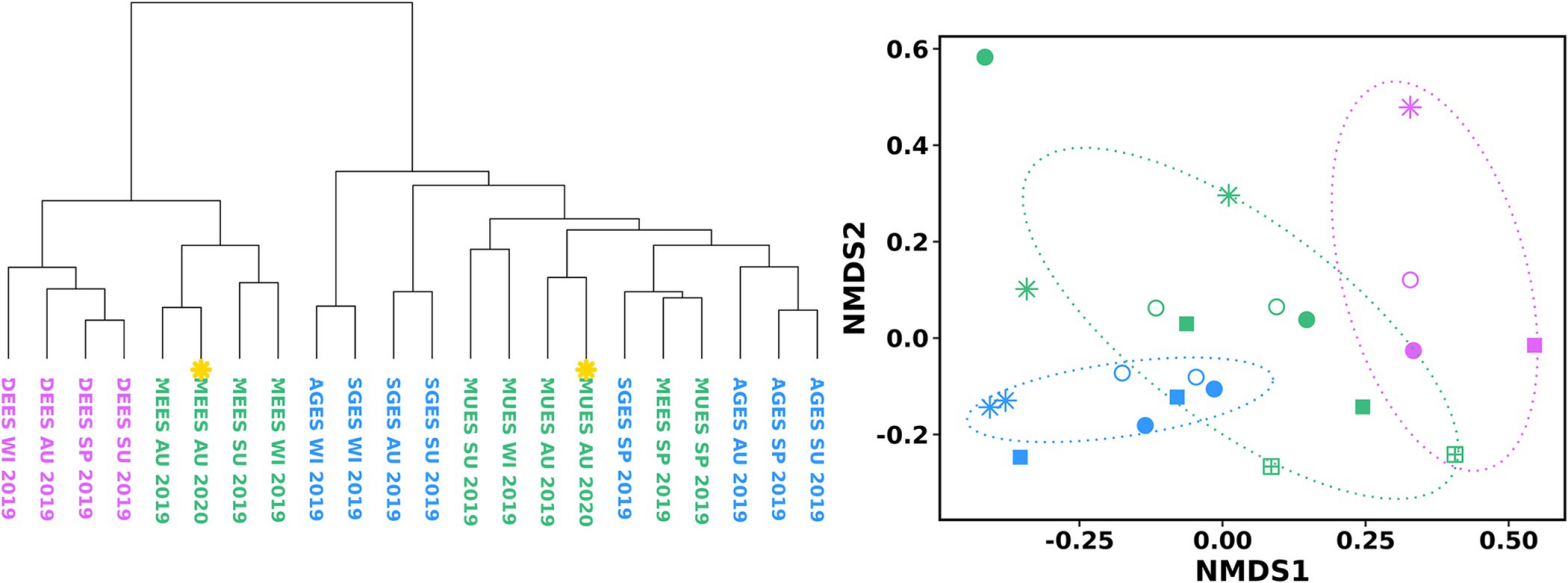
Hierarchical clustering dendrogram (**left**) and NMDS ordination (**right**) of Bray-Curtis dissimilarity matrices based on average relative abundance of mussel gut microbiota at family level. Colours correspond to different harvest regions: Galician region (● AGES + SGES), Basque Country region (● MEES + MUES) and Catalonia region (● DEES). In the left, individuals collected a year later, in 2020, are marked with a yellow asterisk. In the right, shapes of symbols correspond to different harvesting seasons—winter (✳), spring (◯), summer (●), autumn 2019 (◼), autumn 2020 (Σ).

Then, in order to circumvent this issue, we hypothesized whether analysing the overall community structure, instead of specific differences, will be more stable and thus, more suitable as a traceability approach. In this case, a hierarchical clustering based on the overall relative abundance of the bacterial family taxa was performed, by pooling farming site replicates (Fig 6). Results revealed that mussels collected in the Basque Country in two consecutive years clustered together, and even matching the season of harvesting.

## Discussion

### Influence of geographic origin, farm location and season on the digestive microbiota of mussels

The present study investigated the bacterial communities associated to the digestive gland and stomach of *M*. *galloprovincialis* mussels collected seasonally from five different farms located in three regions of Spain. We found that microbial community structure of mussels was significantly different between harvesting locations. Overall, the bacterial composition we found in *Mytilus galloprovincialis* DGS, which was primarily composed of the phyla *Proteobacteria*, *Firmicutes* and *Bacteroidota*, is in concordance with the few previous microbiota studies performed on this species [10, 24–26]. However, none of the previously mentioned studies has analysed the effect of geographical origin on microbial composition as considered here. Still, the influence of the geographic origin in the microbiota of bivalves has previously been described for other bivalves such as oysters and clams [8, 9, 11, 13]. Several factors might have contributed to these changes in the bacterial fingerprints of mussels collected from different geographic locations. Previous studies have demonstrated that bivalve microbiota is influenced by a variety of environmental parameters (i.e. temperature, etc), diet and health condition, which account for the differences between shellfish [10–14].

The present study also showed that bacterial communities present in the digestive gland and stomach of mussels varied seasonally. When individuals from each farm were compared seasonally, it was possible to confirm that bacterial fingerprints associated to the DGS changed significantly. This seasonal variation could be depicted at all taxonomic levels (from OTU to phylum level). These results are in concordance with previous studies showing temporal variability in bivalves microbiota [11, 13, 14].

The seasonal and geographical differences observed in this study are likely reflecting the trends in the surrounding seawater bacterial communities. Mussels collected in this study were not depurated, so they contained a mixture of resident and transient microbiota [27]. As observed in S2 File, the bacterial community profile of mussels and seawater was different, indicating the existence of a host-specific microbiota as previously reported in bivalves [11, 25, 28]. However, the bacterial communities of the seawater and of mussel gut showed somehow a similar clustering pattern, which indicates that the gut microbiota is reflecting the trends of the seawater linked to the geographical origin and season. This result strengthens the potential of using gut microbiota of mussels as a method to trace their geographic origin if mussels are not allowed to depurate transient material prior to gut sampling. Overall, we found that the composition of the microbiota was distinctive between harvesting locations and seasons, with the effect prompted by the origin exceeding the seasonal variability, which is relevant for this approach to be used as an origin traceability tool.

### Is the analysis of the microbiota an accurate tool to trace mussel farming origin?

The present study also assessed the potential of using the bacterial community analysis as an approach to discriminate the geographical origin. Indeed, the results of this preliminary study show that it was possible to discriminate the origin of farmed mussels collected from three regions of the Iberian Peninsula. The differentiation between Catalonian and Galician mussels was very consistent (Fig 2), with samples collected from each of the locations during 2019 clustering in their corresponding group. The bacterial fingerprint of individuals collected within the region of the Basque Country showed a different grouping pattern depending on the taxonomic level. Regarding the stability of the bacterial fingerprints, results have shown that mussel samples collected in two consecutive years clustered together matching even the corresponding season of harvesting (Fig 6).

The present study also allowed to recognize key genera that were potentially relevant to discriminate the geographic origin of the mussels being surveyed, such as, low proportions of *Rubritalea*, Roseibacillus and *Rubripirellula* in Catalonian region and higher levels of *Persicirhabdus* in Galicia. Still, our proposal is to rely on the overall community structure recorded for each production area, using the whole bacterial fingerprint instead of a few markers.

As previously suggested by Liu, Teixeira [8], for this approach to be used as a traceability tool, the development of a database with the reference bacterial fingerprint for each location of interest is necessary. In this way, the bacterial profile of new samples can be compared to this database to verify the origin claimed by the producer or trader. It is also important to highlight that the database must be periodically verified and updated, to enable a successful classification over time. Based on the results obtained in this preliminary study, the time window during which the bacterial fingerprint is likely to remain stable is at least a year, which enables the possibility to rely on previously recorded fingerprints without needing to constantly update the database. A well-designed strategy to maintain the database updated and facilitate the origin classification, could also provide useful information regarding the microbiological safety of the seafood products, allowing to detect potential viral and bacterial pathogens and thus, preventing contaminated products from entering markets.

The microbiota profiling through 16S rRNA amplicon sequencing has shown to have the potential to be used as bioindicator of origin for traceability. This approach could be more suitable than genetic fingerprinting in situations where individuals in from different geographic origins do not form genetically distinct populations [4]. Furthermore, in mussel farms where the spat has been translocated from another region or produced in foreign hatchery, genetic tools would render invalid. In such situations, instead of using genetic tools based on inheritance processes, approaches based on markers reflecting the individuals’ natural surroundings, such as the one described here, could be more appropriate. Still, future analyses should assess how the inter-individual (and inter-population) genetic diversity could shape the bacterial community composition.

### Limitations of the bacterial profiling-based origin traceability approach and potential next steps

Although our results have promising implications for traceability, several limitations were identified. Mussels used in this study were fresh and non-depurated; however, shellfish are usually subjected to depuration, processing and preservation techniques prior to commercialization. These procedures will likely impact the sensitivity of this approach for tracing the geographic origin using 16S rRNA amplicon sequencing of mussel microbiota. Previous studies have reported lower bacterial diversity and richness in depurated [25, 29] and retail [8] bivalves. To fully understand the effect of these techniques in the bacterial fingerprint and to assess the usefulness of this approach in such cases, future studies should follow the methodology described in this study with samples that have been subjected to depuration and other procedures before reaching the final consumers. Besides, this type of bivalves usually has distinct trades since they can be commercialized as fresh, frozen or processed mussel products. Thus, this single methodological approach may not be sufficient to reliably trace the whole international trade. In these cases, a multidisciplinary approach combining different traceability techniques might be the best option.

Another limitation of this study is the restricted number of sampling points analysed and the number of samples used to test the stability of the bacterial fingerprints for one year to another. This prevents a proper understanding of the long-term dynamics and the applicability of this approach in a broader geographical range. Thus, further studies with a more detailed sampling scheme (with additional samples and sampling points where this species is actively cultivated) planned on a longer timeframe are needed.

Besides geographical and seasonal variations, bivalve-associated microbiota is also potentially subjected to short-term significant fluctuations, which may be due to a number of factors, including sudden variations of salinity (e.g. close to estuaries), exceptional summer heatwaves, the presence of algal blooms (also linked with unusual peaks in nutrient availability), etc [10, 12, 28]. The impact of these factors on microbial composition is still poorly understood and the design of this study can not predict whether the occurrence of these events could overcome geographical clustering or not.

Finally, it is also important to keep in mind that, with a broader geographical sampling, the likelihood of overlapping bacterial fingerprints may increase, making the determination of origin based on clustering patterns challenging. Thus, it may be interesting to combine the bacterial profiles obtained with novel machine learning pipelines pipelines (as proposed by Milan, Maroso [7]) to progress towards estimating the assignment probability.

## Supporting information

S1 File Mussel samples used for this study. (XLSX)

S2 File Surface seawater samples. (DOCX)

S3 File Alpha diversity estimators significance. Differences in alpha diversity patterns between sampling points and seasons were analysed using ANOVA and Tukey’s honest significance test of ANOVA (pairwise comparisons) for normally distributed metrics and Kruskal-Wallis and Wilcoxon rank sum test (pairwise comparisons) for non-parametric metrics (“stats”package).
(XLSX)

S4 File Alpha diversity of *M*. *galloprovincialis* DGS microbiota. The box plot shows mean values and standard deviation of the richness, diversity (Simpson and Shannon) and dominance (Berger-Parker) estimators for bacterial communities according to the season and location: in rafts located in estuarine inlets and ports (AGES, SGES, MUES) and in offshore longlines (DEES, MEES).
(DOCX)

S5 FileNMDS ordination based on Bray-Curtis dissimilarities at OTU level of farmed mussel gut microbiota by season.Each symbol represents an individual *M*. *galloprovincialis* mussel; shapes of symbols correspond to different harvesting seasons—winter (✳), spring (◯), summer (●) and autumn (◼)—and colours correspond to different harvest locations in Galician region (● AGES, ● SGES), Catalonia region (● DEES) and Basque Country region (● MEES, ● MUES).(DOCX)

S6 FileNMDS ordination based on Bray-Curtis dissimilarities at OTU level of farmed mussel gut microbiota.Each symbol represents an individual *M*. *galloprovincialis* mussel; shapes of symbols correspond to different harvesting seasons—winter (✳), spring (◯), summer (●), autumn 2019 (◼) and autumn 2019 (Σ)—and colours correspond to different harvest regions: Galician region (● AGES + SGES), Basque Country region (● MEES + MUES) and Catalonia region (● DEES).(DOCX)

S7 FileNMDS ordination based on Bray-Curtis dissimilarities at OTU level of farmed mussel gut microbiota by sampling point.Each symbol represents an individual *M*. *galloprovincialis* mussel; shapes of symbols correspond to different harvesting seasons—winter (✳), spring (◯), summer (●) and autumn (◼)—and colours correspond to different harvest locations in Galician region (● AGES, ● SGES), Catalonia region (● DEES) and Basque Country region (● MEES, ● MUES).(DOCX)

S8 FileRelative abundance of genus that differed significantly (Kruskall Wallis, p<0.05) between the five mussel farms.Points are median values; lines represent the interquartile range and the black vertical line is the limit of detection. Taxa with a median relative abundance < 0.5% for all the locations were grouped in “Others”. Colours correspond to different harvest locations in Galician region (● AGES, ● SGES), Catalonia region (● DEES) and Basque Country region (● MEES, ● MUES).(DOCX)

S9 File Relative abundances at genus level; 2019 vs 2020. (A). Relative abundance of bacterial communities, at genus level, of mussel DGS harvested from five different farms during 2019 and 2020: in Galician region (AGES, SGES), Catalonia region (DEES) and Basque Country region (MEES, MUES). Taxa not within the 20 most abundant families were pooled together as “Other”. (B). Relative abundance of genus that differed significantly (Kruskall Wallis, p<0.05) between the mussel farms in 2019 and 2020. Points are median values; lines represent the interquartile range and the black vertical line is the limit of detection. Taxa with a median relative abundance < 0.5% for all the locations were grouped in “Others”.
(DOCX)
