# Supplementary material for: Gut microbiota fingerprinting as a potential tool for tracing the geographical origin of farmed mussels (Mytilus galloprovincialis)
Source: PLoS One. 2023 Aug 30;18(8):e0290776. doi: 10.1371/journal.pone.0290776 (PMC10468044; doi:10.1371/journal.pone.0290776)
Supplement: S2 File — (DOCX) [file pone.0290776.s002.docx]

**Supplementary material 2.** Surface seawater samples.

Materials and methods:

As a control, surface seawater samples (2 L per location/season) were also collected during 2019 using sterile plastic bottles. Water samples (n=20) were pre‐filtered through 3 μm GF/A Whatman filters (Whatman, UK) and filtered onto 0.2 μm Millipore Sterivex GP capsule filters (Millipore, USA). Filtered DNA extraction was performed following the protocol described by Spens, Evans et al. (2017) in Appendix S1, using DNeasy Blood & Tissue Kit (Qiagen, Germany). Extracted DNA concentration was determined by means of the Quant-iT dsDNA HS assay kit using a Qubit® 1.0 Fluorometer (Life Technologies, USA). The 16S amplicon library of water samples were prepared and sequenced together with 2019 mussel samples. Sequences were analysed following the same procedure as for mussels.

Results:

**Figure A**. Hierarchical clustering dendrogram based on Bray-Curtis dissimilarities at OTU level of both water and farmed mussel gut microbiota. Each label represents a unique sample; collected in different harvesting seasons - winter (WI), spring (SP), summer (SU), autumn 2019 (AU), autumn 2020 AU20) - and colours correspond to different harvest locations of mussels in Galician region (*●* AGES, ● SGES), Catalonia region (● DEES) and Basque Country region (● MEES, ● MUES) and water samples (●).


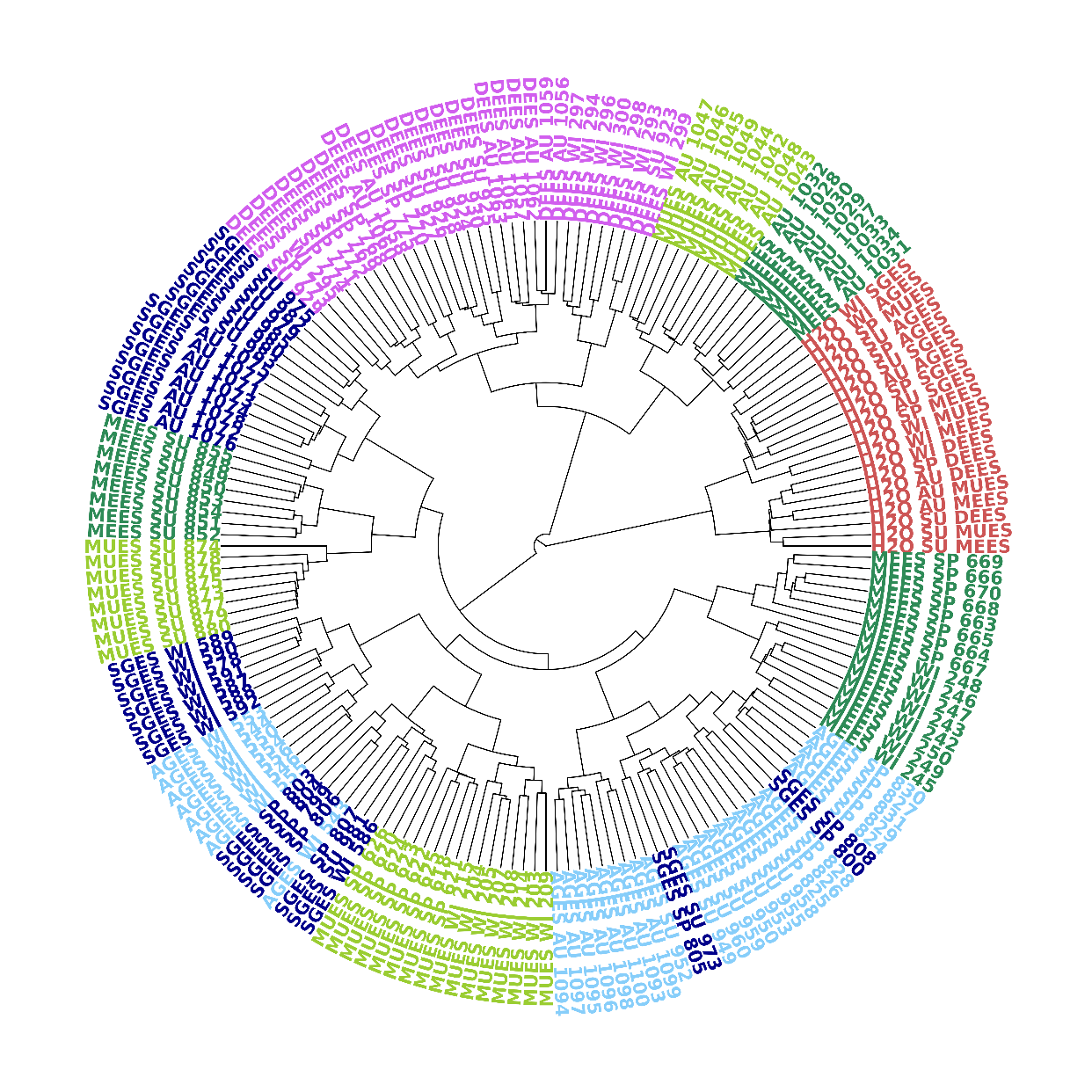


**Figure B**. Hierarchical clustering dendrogram based on Bray-Curtis dissimilarities at OTU level of only water samples. Each label represents a unique sample; collected in different harvesting seasons - winter (WI), spring (SP), summer (SU), autumn 2019 (AU), autumn 2020 AU20) - and colours correspond to different harvest locations of surface seawater in Galician region (*●* AGES, ● SGES), Catalonia region (● DEES) and Basque Country region (● MEES, ● MUES).


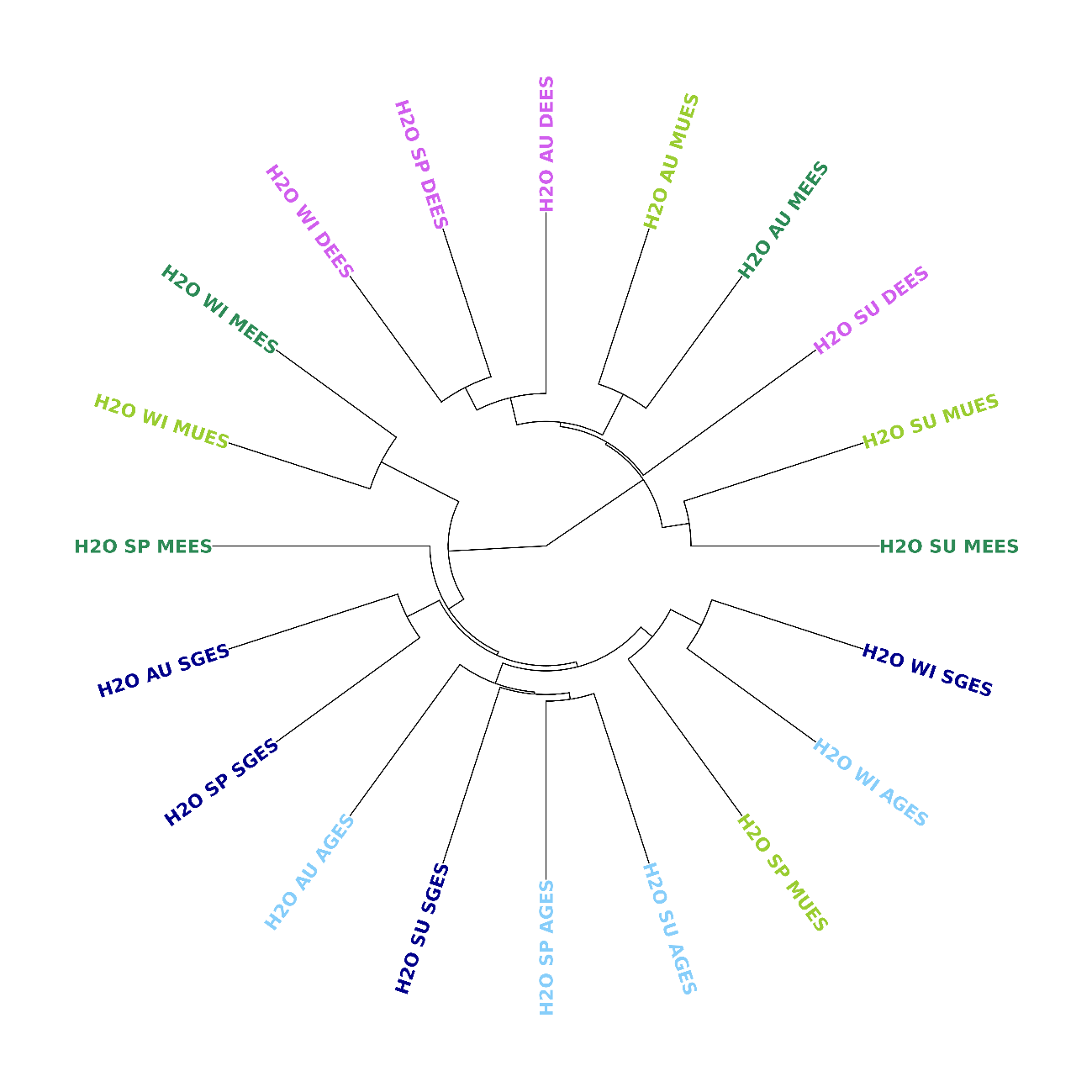


Spens, J., et al. (2017). "Comparison of capture and storage methods for aqueous macrobial eDNA using an optimized extraction protocol: advantage of enclosed filter." Methods in Ecology and Evolution **8**(5): 635-645.
