# Supplementary material for: Gut microbiota fingerprinting as a potential tool for tracing the geographical origin of farmed mussels (Mytilus galloprovincialis)
Source: PLoS One. 2023 Aug 30;18(8):e0290776. doi: 10.1371/journal.pone.0290776 (PMC10468044; doi:10.1371/journal.pone.0290776)

**Supplementary material 4.** Alpha diversity of *M. galloprovincialis* DGS microbiota. The box plot shows mean values and standard deviation of the richness, diversity (Simpson and Shannon) and dominance (Berger-Parker) estimators for bacterial communities according to the season and location: in rafts located in estuarine inlets and ports (AGES, SGES, MUES) and in offshore longlines (DEES, MEES).


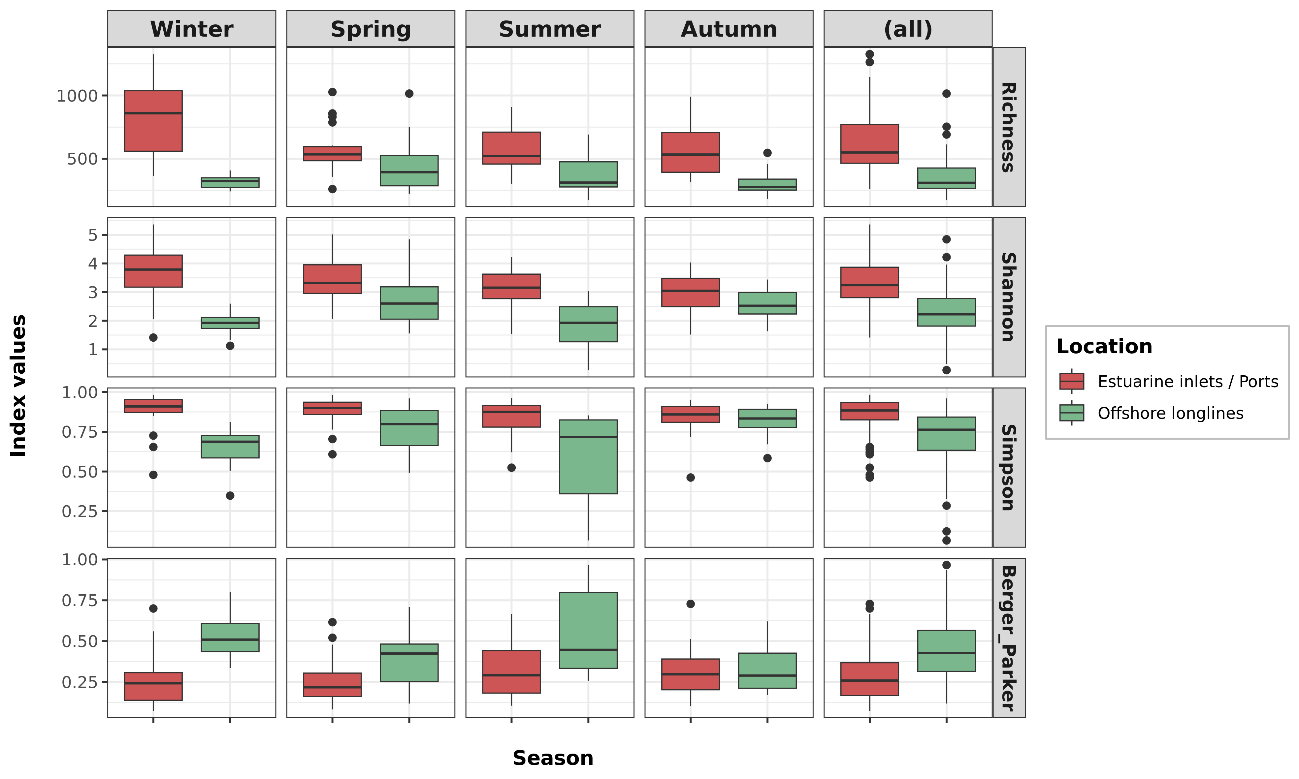

Supplement: S4 File — The box plot shows mean values and standard deviation of the richness, diversity (Simpson and Shannon) and dominance (Berger-Parker) estimators for bacterial communities according to the season and location: in rafts located in estuarine inlets and ports (AGES, SGES, MUES) and in offshore longlines (DEES, MEES). (DOCX) [file pone.0290776.s004.docx]
