# Supplementary material for: Gut microbiota fingerprinting as a potential tool for tracing the geographical origin of farmed mussels (Mytilus galloprovincialis)
Source: PLoS One. 2023 Aug 30;18(8):e0290776. doi: 10.1371/journal.pone.0290776 (PMC10468044; doi:10.1371/journal.pone.0290776)

**Supplementary material 5.** NMDS ordination based on Bray-Curtis dissimilarities at OTU level of farmed mussel gut microbiota by season. Each symbol represents an individual *M. galloprovincialis* mussel; shapes of symbols correspond to different harvesting seasons - winter (🞽), spring (⭘), summer (⚫) and autumn (◾) - and colours correspond to different harvest locations in Galician region (*●* AGES, ● SGES), Catalonia region (● DEES) and Basque Country region (● MEES, ● MUES).


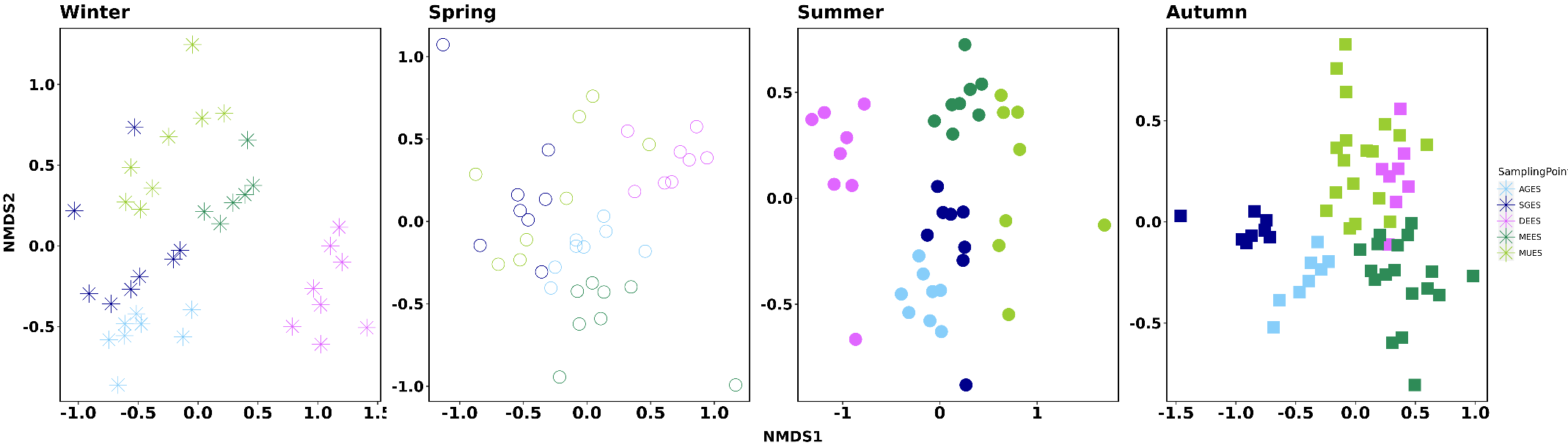

Supplement: S5 File — Each symbol represents an individual M. galloprovincialis mussel; shapes of symbols correspond to different harvesting seasons—winter (✳), spring (◯), summer (●) and autumn (◼)—and colours correspond to different harvest locations in Galician region (● AGES, ● SGES), Catalonia region (● DEES) and Basque Country region (● MEES, ● MUES). (DOCX) [file pone.0290776.s005.docx]
