# Supplementary material for: Gut microbiota fingerprinting as a potential tool for tracing the geographical origin of farmed mussels (Mytilus galloprovincialis)
Source: PLoS One. 2023 Aug 30;18(8):e0290776. doi: 10.1371/journal.pone.0290776 (PMC10468044; doi:10.1371/journal.pone.0290776)

**Supplementary material 6.** NMDS ordination based on Bray-Curtis dissimilarities at OTU level of farmed mussel gut microbiota. Each symbol represents an individual *M. galloprovincialis* mussel; shapes of symbols correspond to different harvesting seasons - winter (🞽), spring (⭘), summer (⚫), autumn 2019 (◾) and autumn 2019 (⮽) - and colours correspond to different harvest regions: Galician region (● AGES + SGES), Basque Country region (● MEES + MUES) and Catalonia region (● DEES).


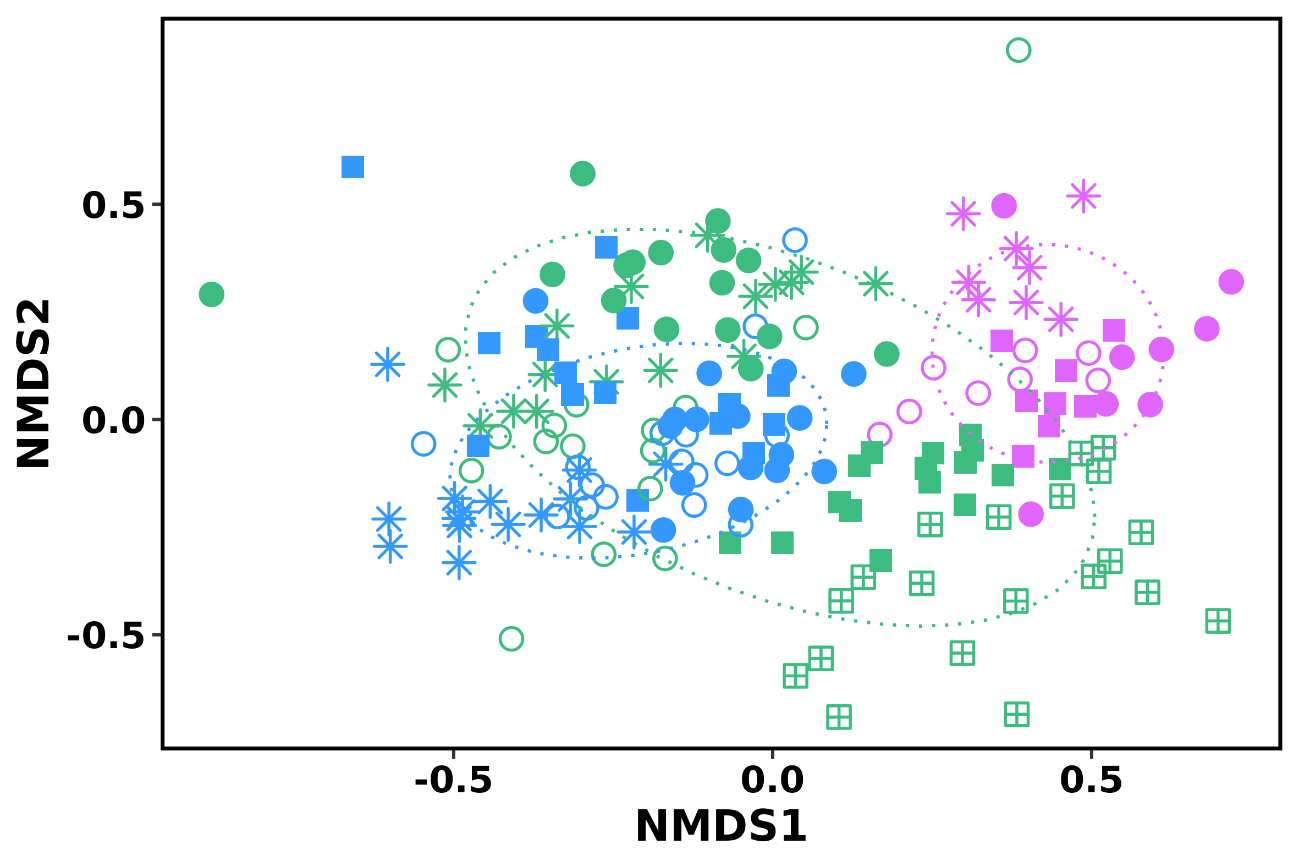

Supplement: S6 File — Each symbol represents an individual M. galloprovincialis mussel; shapes of symbols correspond to different harvesting seasons—winter (✳), spring (◯), summer (●), autumn 2019 (◼) and autumn 2019 (Σ)—and colours correspond to different harvest regions: Galician region (● AGES + SGES), Basque Country region (● MEES + MUES) and Catalonia region (● DEES). (DOCX) [file pone.0290776.s006.docx]
