# Supplementary material for: Gut microbiota fingerprinting as a potential tool for tracing the geographical origin of farmed mussels (Mytilus galloprovincialis)
Source: PLoS One. 2023 Aug 30;18(8):e0290776. doi: 10.1371/journal.pone.0290776 (PMC10468044; doi:10.1371/journal.pone.0290776)
Supplement: S8 File — Points are median values; lines represent the interquartile range and the black vertical line is the limit of detection. Taxa with a median relative abundance < 0.5% for all the locations were grouped in “Others”. Colours correspond to different harvest locations in Galician region (● AGES, ● SGES), Catalonia region (● DEES) and Basque Country region (● MEES, ● MUES). (DOCX) [file pone.0290776.s008.docx]

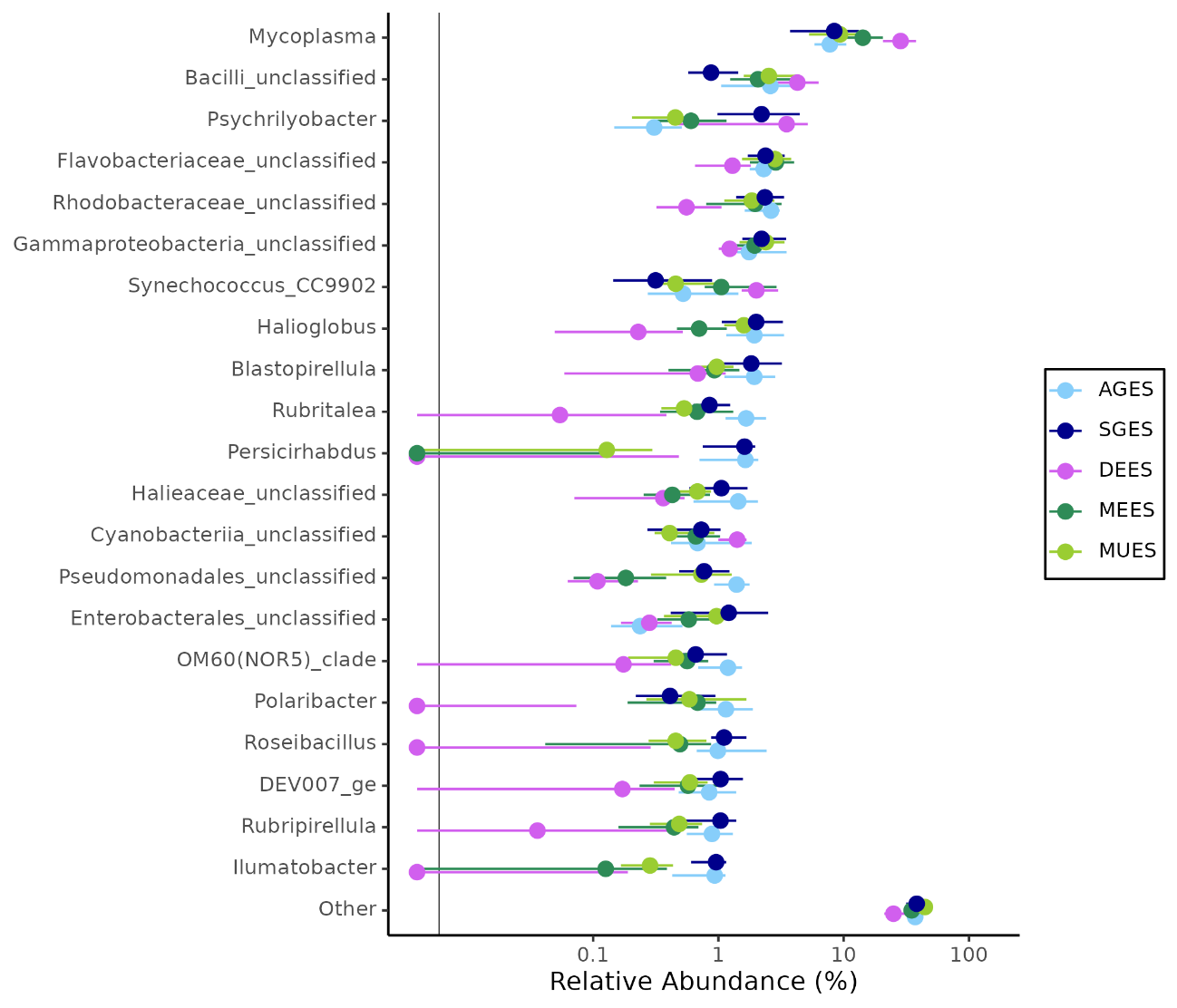


**Supplementary material 8.** Relative abundance of genus that differed significantly (Kruskall Wallis, p<0.05) between the five mussel farms. Points are median values; lines represent the interquartile range and the black vertical line is the limit of detection. Taxa with a median relative abundance < 0.5% for all the locations were grouped in “Others”. Colours correspond to different harvest locations in Galician region (*●* AGES, ● SGES), Catalonia region (● DEES) and Basque Country region (● MEES, ● MUES).
