# Supplementary material for: Gut microbiota fingerprinting as a potential tool for tracing the geographical origin of farmed mussels (Mytilus galloprovincialis)
Source: PLoS One. 2023 Aug 30;18(8):e0290776. doi: 10.1371/journal.pone.0290776 (PMC10468044; doi:10.1371/journal.pone.0290776)
Supplement: S9 File — (A). Relative abundance of bacterial communities, at genus level, of mussel DGS harvested from five different farms during 2019 and 2020: in Galician region (AGES, SGES), Catalonia region (DEES) and Basque Country region (MEES, MUES). Taxa not within the 20 most abundant families were pooled together as “Other”. (B). Relative abundance of genus that differed significantly (Kruskall Wallis, p<0.05) between the mussel farms in 2019 and 2020. Points are median values; lines represent the interquartile range and the black vertical line is the limit of detection. Taxa with a median relative abundance < 0.5% for all the locations were grouped in “Others”. (DOCX) [file pone.0290776.s009.docx]

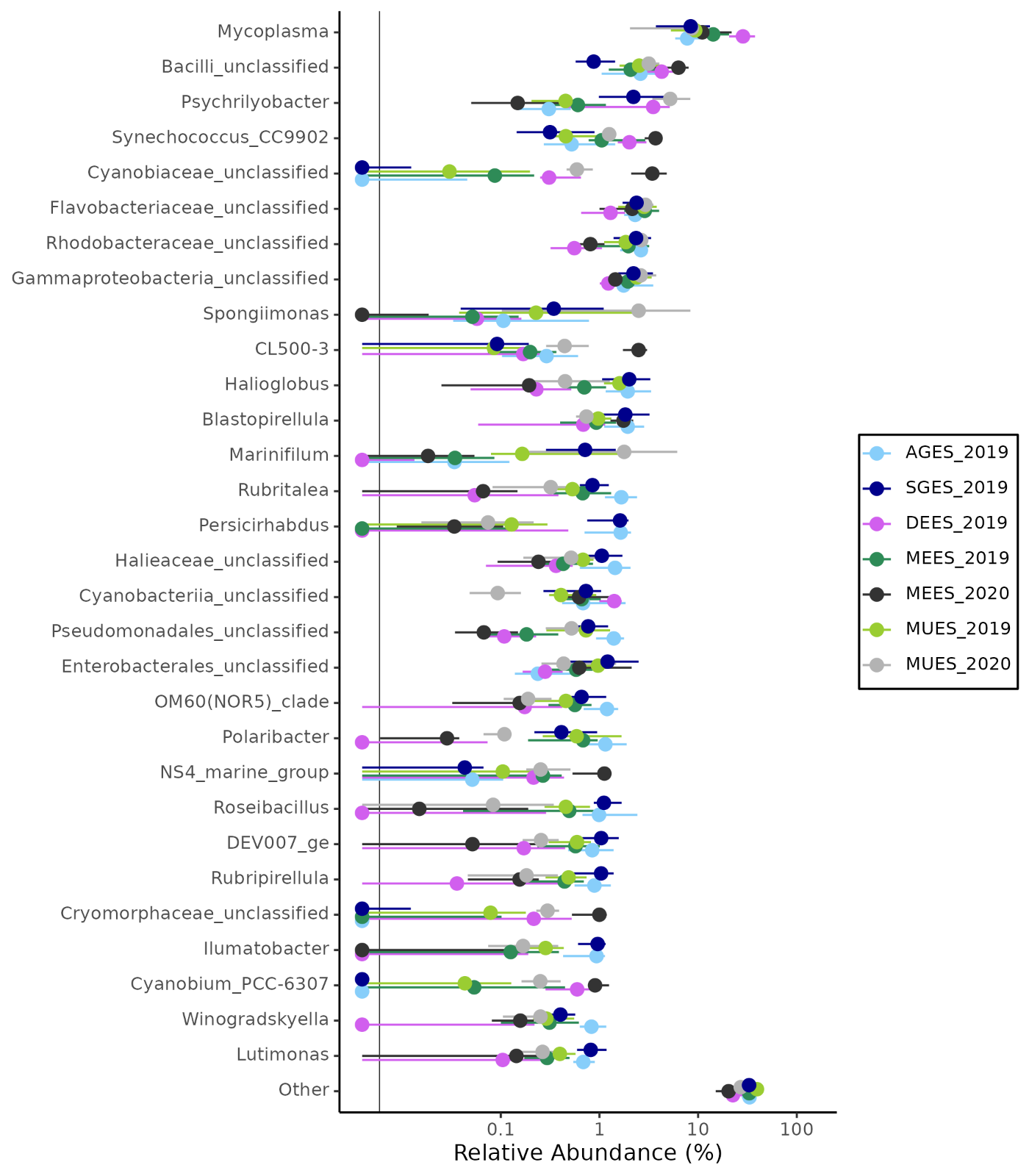

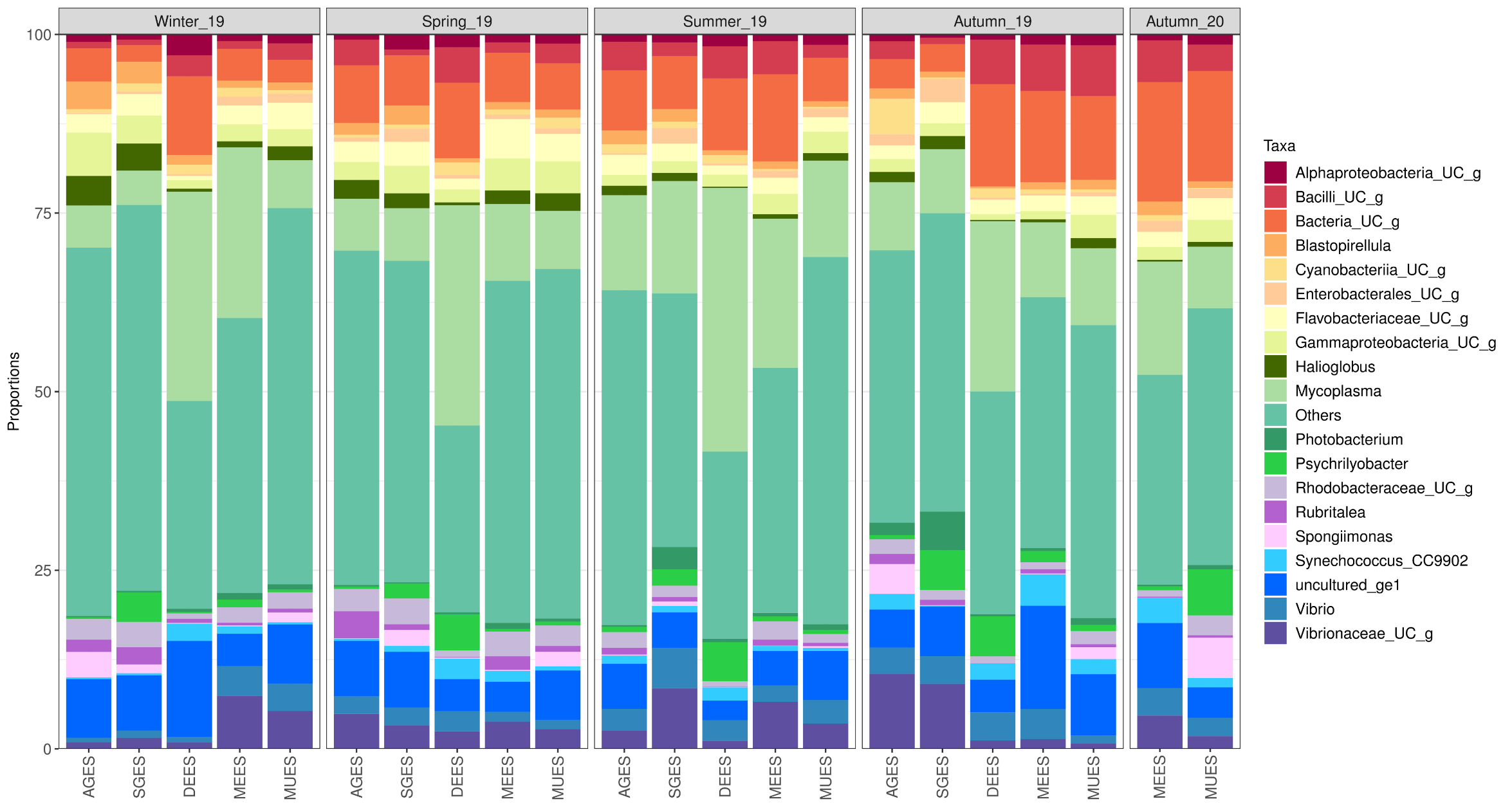


**Supplementary material 9**. Relative abundances at genus level; 2019 vs 2020.

**Page 1.** Relative abundance of genus that differed significantly (Kruskall Wallis, p<0.05) between the mussel farms in 2019 and 2020. Points are median values; lines represent the interquartile range and the black vertical line is the limit of detection. Taxa with a median relative abundance < 0.5% for all the locations were grouped in “Others”.

**Page 2.** Relative abundance of bacterial communities, at genus level, of mussel DG harvested from five different farms during 2019 and 2020: in Galician region (AGES, SGES), Catalonia region (DEES) and Basque Country region (MEES, MUES). Taxa not within the 20 most abundant families were pooled together as “Other”.
